# Influences of Chloride and Hypochlorite on Neutrophil Extracellular Trap Formation

**DOI:** 10.1371/journal.pone.0042984

**Published:** 2012-08-13

**Authors:** Kathryn Akong-Moore, Ohn A. Chow, Maren von Köckritz-Blickwede, Victor Nizet

**Affiliations:** 1 Department of Pediatrics, University of California San Diego, La Jolla, California, United States of America; 2 Skaggs School of Pharmacy and Pharmaceutical Sciences, University of California San Diego, La Jolla, California, United States of America; 3 Rady Children’s Hospital, San Diego, California, United States of America; Dr. Margarete Fischer-Bosch and University of Tübingen, Germany

## Abstract

**Background:**

The release by neutrophils of DNA-based extracellular traps (NETs) is a recently recognized innate immune phenomenon that contributes significantly to control of bacterial pathogens at tissue foci of infection. NETs have also been implicated in the pathogenesis of non-infectious diseases such as small vessel vasculitis, lupus and cystic fibrosis lung disease. Reactive oxygen species (ROS) are important mediators of NET generation (NETosis). Neutrophils with reduced ROS production, such as those from patients with chronic granulomatous disease or myeloperoxidase (MPO) deficiency, produce fewer NETs in response to inflammatory stimuli. To better understand the roles of various ROS in NETosis, we explore the role of MPO, its substrates chloride ion (Cl^−^) and hydrogen peroxide (H_2_O_2_), and its product hypochlorite (HOCl) in NETosis.

**Findings:**

In human peripheral blood neutrophils, pharmacologic inhibition of MPO decreased NETosis. Absence of extracellular Cl^−^, a substrate for MPO, also reduced NETosis. While exogenous addition of H_2_O_2_ and HOCl stimulated NETosis, only exogenous HOCl could rescue NETosis in the setting of MPO inhibition. Neither pharmacological inhibition nor genetic deletion of MPO in murine neutrophils blocked NETosis, in contrast to findings in human neutrophils.

**Conclusions:**

Our results pinpoint HOCl as the key ROS involved in human NETosis. This finding has implications for understanding innate immune function in diseases in which Cl^−^ homeostasis is disturbed, such as cystic fibrosis. Our results also reveal an example of significant species-specific differences in NET phenotypes, and the need for caution in extrapolation to humans from studies of murine NETosis.

## Introduction

When epithelial barriers are breached by microbial pathogens, a critical first responder of the innate immune response is the neutrophil. Preloaded with a potent antimicrobial armamentarium, neutrophils are activated to kill pathogens through phagocytosis, generation of reactive oxygen and nitrogen species, and release of antimicrobial peptides and proteases into the extracellular space [1]. Another more recently described mechanism of microbial killing by neutrophils at the site of infection is the formation of neutrophil extracellular traps (NETs), the end-product of a distinct form of programmed cell death termed “NETosis” [2], [3], [4].

The morphologic changes of NETosis have been observed in detail through electron and immunofluorescence microscopy. Briefly, nuclear chromatin decondensation occurs, and the characteristic multi-lobulated appearance is lost. Nuclear membrane disruption then leads to mixing of decondensed chromatin with cytoplasmic granule proteins. Finally, cell membrane disruption occurs, and the intracellular contents are expelled in a web-like structure in the extracellular space. The resulting NETs are composed of a meshwork of decondensed chromatin DNA filaments, covered in various antimicrobial cellular components, such as histones, anti-microbial peptides (AMPs), proteases, and enzymes such as myeloperoxidase (MPO) that produce toxic reactive oxygen species (ROS). NETs have been established to trap and kill bacteria and fungi *in vitro*, and to have an important role in innate immune function *in vivo*.

The MPO enzyme itself is implicated in the generation of NETs, since neutrophils isolated from patients with MPO deficiency have markedly reduced NET formation in response to inflammatory stimuli [5]. MPO catalyzes the production of hypochlorite (HOCl), one of the most potent neutrophil ROS [6], using hydrogen peroxide (H_2_O_2_) and chloride (Cl^−^) as substrates. Recently, the availability of Cl^−^ in the extracellular medium was shown to be important in neutrophil killing of the bacterial pathogen *Pseudomonas aeruginosa* [7]. One mechanism supported by these data was that MPO catalyzed production of HOCl within the phagolysosome, after Cl^−^ was transported to this compartment by the cystic fibrosis transmembrane regulator (CFTR) ion channel. With the new knowledge that MPO is an important upstream regulator of NETosis [5], we hypothesized that perhaps the bacterial killing defect seen in the absence of extracellular Cl^−^ was also the result of impaired NETosis. Thus in the present work, we sought to explore the role of MPO, extracellular chloride and HOCl in NET generation in human and mouse neutrophils.

## Results

**Figure 1:**
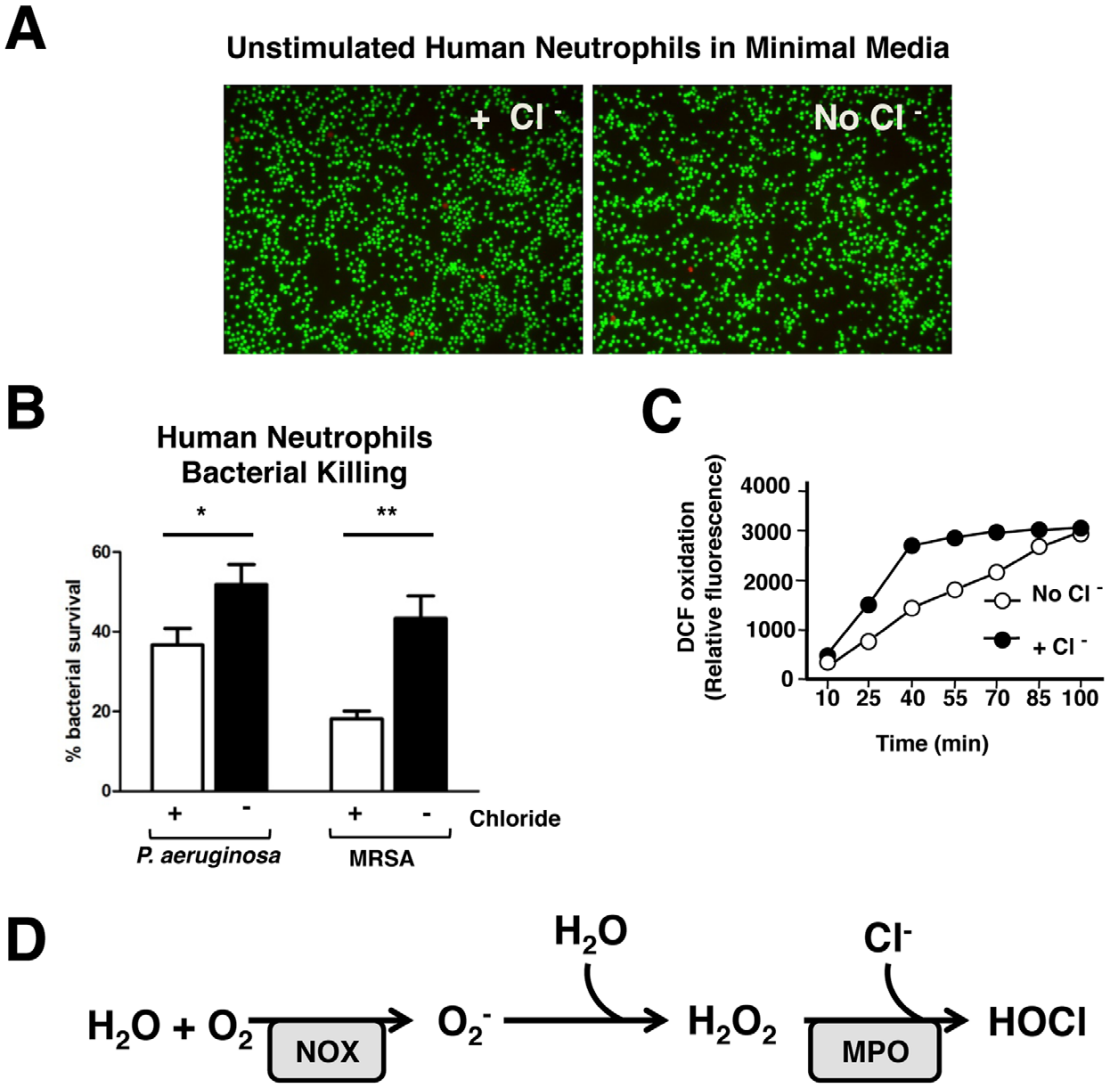
Extracellular chloride (Cl^−^) contributes to bacterial killing by human neutrophils. (A) Human peripheral neutrophils were incubated in minimal media with or without Cl^−^ for 3 h (2×10^6^ cells/well). Cells were then assayed for viability using calcein (green) which identifies live cells, and ethidium homodimer (red) which binds DNA but cannot cross intact cell membranes, thus staining dead cells. Neutrophils incubated for 3 h in minimal media with and without Cl^−^ are equally viable. (n = 3 wells per condition) (B) Human peripheral neutrophils were incubated in minimal media with or without Cl^−^. Bacteria were incubated in same media, with and without neutrophils (MOI  = 0.1). Bacterial killing by neutrophils was reduced in the absence of extracellular Cl^−^. Each condition performed in triplicate. (C) Oxidative burst was assayed using intracellular DCF dye that fluoresces when oxidized. The production of oxidants was slower in the absence of Cl^−^, but similar peak production of oxidants was achieved by 100 min in the presence or absence of Cl^−^. Each condition was done in triplicate in each experiment (D) Oxidant production in activated neutrophils occurs in a series of reactions. Enzymes known to be important in these reactions, such as NADPH oxidase and MPO are also essential for NETosis.

As reported by Painter et al., human neutrophil killing was decreased in the absence of Cl^−^ in the extracellular medium. Using their published methods, we made minimal media that are osmotically identical, but differ in chloride content, by replacing chloride with gluconate. We first verified that neutrophils are equally viable in both these media, using staining for live and dead cells (**Figure 1a**). We also confirmed in our assay system using *Pseudomonas aeruginosa* strain PAO1, and methicillin-resistant *Staphylococcus aureus* strain UAMS 1182 that neutrophils in minimal media lacking Cl^−^ do indeed have defects in bacterial killing (**Figure 1b**). Neutrophils can also kill pathogens by producing and releasing toxic ROS via a process called “oxidative burst”. Oxidative burst activity was assayed in the presence and absence of extracellular Cl^−^ in minimal media. When Cl^−^ is absent, the kinetics of the neutrophil oxidative burst were slower than when Cl^−^ was present; however, the peak of superoxide generation was ultimately equivalent by 100 min in the presence or absence of Cl^−^ (**Figure 1c**). The production of ROS by neutrophils via oxidative burst (**Fig. 1d**) is known to be critical in the generation of NETs, as neutrophils from patients with defects in assembly of a functional NADPH oxidase complex are unable to generate NETs. Since Cl^−^ levels did not significantly affect oxidative burst, we hypothesized that perhaps the role of Cl^−^ in bacterial killing may in part be due to a role in NET generation.

**Figure 2:**
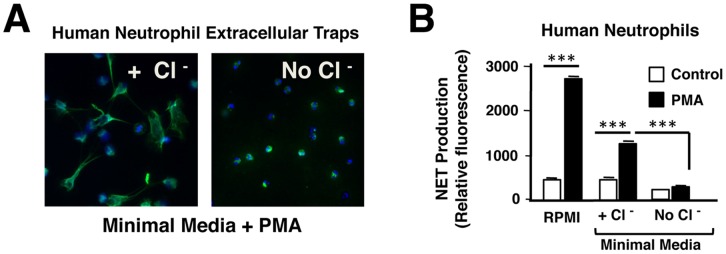
NET formation requires extracellular chloride (Cl^−^). Human peripheral neutrophils (2×10^6^ cells/well) were incubated in minimal media with or without extracellular Cl^−^, and stimulated to form NETs with PMA for 3 h. NETosis was completely inhibited in the absence of extracellular Cl^−^, as seen by (A) immunofluorescence using DAPI to counterstain DNA (blue) and anti-MPO antibody (green) to visualize NETs, and (B) quantification of released extracellular DNA (each condition done in triplicate).

To now explore the role of Cl^−^ in NET formation, we performed phorbol 12-myristate 13-acetate (PMA)-stimulated NET assays at 3 h in the presence and absence of Cl^−^ in the minimal culture medium. At this time point, NETosis is maximal when neutrophils are stimulated with PMA. Using immunostaining and microscopy, NETosis was clearly decreased in the absence of Cl^−^ (**Figure 2a**). This finding was confirmed using a quantitative assay for extracellular DNA release, which showed negligible release in the absence of extracellular Cl^−^ (**Figure 2b**). These data suggest that levels of extracellular Cl^−^ in the milieu in which a neutrophil is activated, can affect its ability to kill bacteria by affecting NET formation.

**Figure 3:**
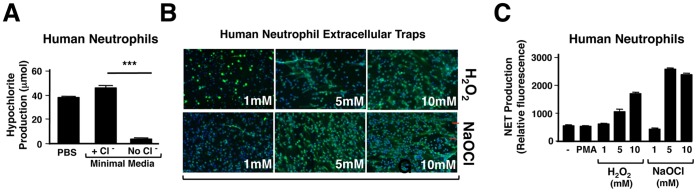
Human neutrophils form NETs in response to exogenous oxidants. (A) Hypochlorite (HOCl) release by activated neutrophils incubated in minimal media with or without Cl^−^ was measured using a colorimetric assay for chlorination of extracellular taurine. Production of HOCl by activated neutrophils requires extracellular Cl^−^. Each condition was done in triplicate. (B,C) NETosis increased in dose-dependent manner in response to exogenous hydrogen peroxide and sodium HOCl, as visualized using immunofluorescence and microscopy (blue: DAPI, green: anti-MPO antibody) (B), and using quantification of released extracellular DNA (C) as assays of NETosis. Neutrophils were plated at 2×10^6^ cells/well.

Superoxide produced by NADPH oxidase can rapidly dismutate to form H_2_O_2_. H_2_O_2_ and Cl^−^ ions are substrates for a reaction catalyzed by MPO, to produce HOCl (**Figure 1c**). We hypothesized that inhibition of NETosis in the absence of Cl^−^ was due to substrate deprivation and a lack of HOCl production catalyzed by MPO. Using a sensitive assay of HOCl production, and hence MPO activity, described by Kettle et al. [8], we confirmed that in the absence of extracellular Cl^−^, HOCl production by human neutrophils is negligible (**Figure 3a**). In our proposed model, HOCl is the key ROS species in NETosis, and MPO and its substrates H_2_O_2_ and Cl^−^ are required for NETosis in order to produce HOCl. If this model is correct, addition of exogenous NaOCl alone should stimulate NET formation. To test this hypothesis, we compared the addition of exogenous H_2_O_2_ vs. exogenous NaOCl to PMA-stimulated PMNs cultured in RPMI media containing physiologic levels of Cl^−^. Both H_2_O_2_ and NaOCl stimulated NETosis that was much more rapid (observed within 30 min) than PMA stimulation alone (**Figure 3b, c**). These effects were concentration-dependent, and support our hypothesis that HOCl is an important molecular signal contributing to NET generation.

**Figure 4:**
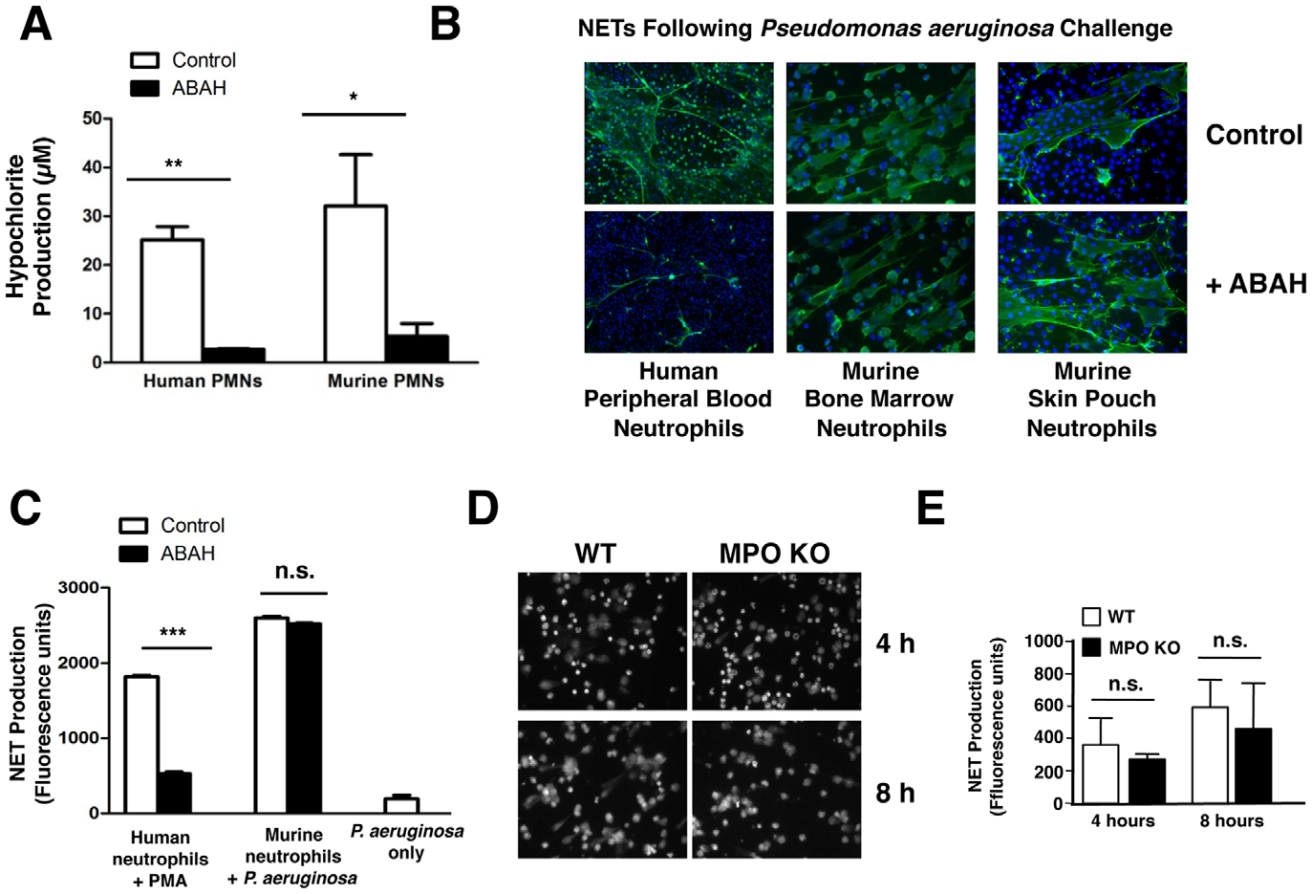
Differing roles of myeloperoxidase (MPO) in human and murine NETosis. (A) The pharmacological agent ABAH inhibited MPO in both human, and murine bone marrow-derived neutrophils, as evidenced by significantly decreased MPO-catalyzed HOCl release after activation with PMA. All conditions were done in triplicate. (B,C) Human peripheral neutrophils were stimulated with potent trigger for NETosis (live *Pseudomonas aeruginosa* bacteria, MOI  = 1) in the presence of ABAH or vehicle control (blue: DAPI, green: anti-H2A/H2B/DNA complex antibody). Inhibition of MPO with ABAH almost completely blocked human NET formation even in response to potent stimuli (B, upper panels). However, inhibition of MPO with ABAH in murine bone marrow-derived neutrophils (B, middles panels) or skin pouch derived neutrophils (B, lower panels) did not block NET formation significantly. Data were obtained using (B) microscopy and immunofluorescence and (C) quantification of released extracellular DNA. As *P. aeruginosa* is known to release extracellular DNA itself, we included a bacteria only control, showing that bacteria alone do not significantly contribute to the elevation in released extracellular DNA. All conditions were performed in triplicate. (D,E) Bone marrow derived-neutrophils from MPO knockout mice or wild type siblings were stimulated for indicated times with live *P. aeruginosa* bacteria (MOI  = 1). No differences in NET formation were observed with either direct visualization with (D) immunofluorescence and microscopy or (E) quantification of released extracellular DNA. All conditions done in triplicate, experiment repeated two independent times.

**Figure 5:**
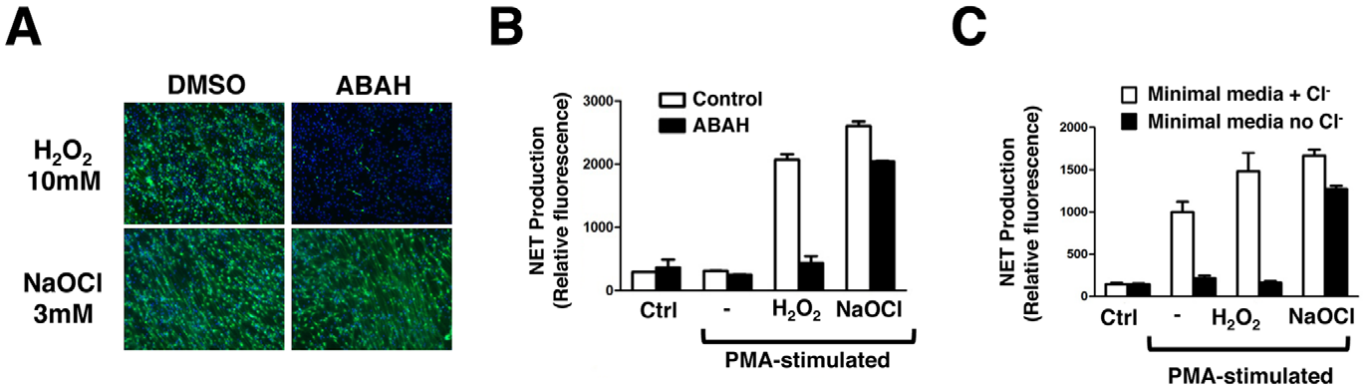
Key role of hypochlorite (HOCl) in triggering NETs. (A,B) Human neutrophils (2×10^6^ cells/well) were pre-treated with ABAH, and then stimulated with either H_2_O_2_ or NaOCl. MPO inhibition blocked H_2_O_2_-stimulated but not NaOCl-stimulated NETosis, as seen with (A) microscopy and (B) quantification of extracellular DNA release, indicating that HOCl is the central oxidant species that triggers NET formation in human neutrophils. All conditions were done in triplicate. (C) Stimulated human neutrophils incubated in minimal media with or without Cl^−^ were treated with exogenous H_2_O_2_ or NaOCl at indicated concentrations. In the absence of Cl^−^, human NETosis was rescued by addition of exogenous NaOCl, but not by H_2_O_2_, again illustrating that HOCl represents the central oxidant species for NET formation. All conditions were done in triplicate.

In humans, deficiency in MPO is associated with decreased NET production [5], and the MPO enzyme is also an important structural component of NETs [9]. Earlier investigations have suggested that perhaps the enzymatic activity of MPO is not essential to its role in NETosis [10]. To address this question directly, we used the specific inhibitor 4-amino benzoic acid hydrazide (ABAH), which potently inhibits MPO enzymatic activity [11]. We confirmed that ABAH is very effective in blocking HOCl generation in both human and murine neutrophils (**Figure 4a**). Inhibition of MPO with ABAH nearly completely blocked NETosis in response to a very potent trigger, live *P. aeruginosa* bacteria, in human peripheral blood neutrophils (**Figure 4b** **, upper panels**), demonstrating that the enzymatic activity of MPO is required for NETosis. Unexpectedly, murine bone marrow-derived neutrophils stimulated with PMA and live *P. aeruginosa* bacteria were still able to form NETs despite effective MPO inhibition with ABAH (**Figure 4b** **, middle panels**). This observed difference in NETosis with MPO inhibition between human and murine neutrophils could be attributable to differences in maturity of the cells being used (human peripheral blood neutrophils vs. murine bone marrow-derived neutrophils. To address this point, we obtained mature murine neutrophils by recruitment to a subdermal skin pouch following injection of LPS into the pouch. These neutrophils are recruited from peripheral blood, and thus similar in maturity to the human neutrophils from peripheral blood. Stimulation of murine skin pouch neutrophils with live *P. aeruginosa* bacteria in the setting of inhibition of MPO with ABAH again did not lead to decreased NETosis (**Figure 4b** **, lower panels**). To corroborate this finding, we compared the behavior of neutrophils from wild-type (WT) control mice and knockout mice harboring a deletion of the MPO gene [12]. This was done using bone marrow-derived neutrophils, since access to live mice for skin pouch experiments was not possible. When NET formation was assayed using both immunostaining and microscopy (**Figure 4c**) and quantification of released DNA (**Figure 4d**), bone marrow-derived neutrophils from WT and MPO knockout mice produced NETs at the same frequency. The combined pharmacologic and genetic studies may indicate a difference in the role of MPO-catalyzed HOCl production between species, as the enzyme is essential for efficient NETosis in humans but appears to be dispensible in the murine model. To probe further how HOCl can serve as a central regulator of human NETosis, we tested whether exogenous NaOCl was sufficient to rescue NETosis when MPO is inhibited pharmacologically by ABAH. Human peripheral blood neutrophils were incubated in RPMI in the presence or absence of ABAH, and then stimulated with PMA either in the presence or absence of H_2_O_2_ or NaOCl for only 1 h. This shorter incubation time is not sufficient to provoke NETosis in PMA only-treated cells. While H_2_O_2_ was unable to rescue NETosis in the setting of MPO inhibition, NaOCl fully rescued NET production to levels similar to those observed with uninhibited control neutrophils (**Figure 5a,b**). Furthermore, when NETosis is suppressed by depletion of extracellular Cl^−^, as demonstrated in **Figure 2**, only the addition of NaOCl and not H_2_O_2_ is able to rescue NET formation (**Figure 5c**), consistent with the unique importance of HOCl in this human neutrophil activation pathway.

## Discussion

Our results indicate that the important requirement of extracellular Cl^−^ for efficient neutrophil bacterial killing involves not only phagolysosomal function [7], but may also extends to the extracellular killing mechanism of NETosis. The contribution of NETosis to overall neutrophil bacterial killing is still actively under investigation in many laboratories, and the study of isolated NET-mediated bacterial killing is fundamentally dependent on the specific conditions used. We observed that Cl^−^ depletion or MPO inhibition decreases human NETosis, that exogenous NaOCl stimulates NETs in a dose-dependent manner, and that addition of NaOCl can rescue the NET formation defect when MPO is pharmacologically inhibited. In the physiologic situation *in* *vivo*, MPO and HOCl are likely to function primarily in a cell autonomous fashion, since stimulated neutrophils release HOCl only in the micromolar range [8], and WT neutrophils are not able to rescue the NET phenotype of MPO-deficient neutrophils under co-culture conditions [5].

The role of extracellular Cl^−^ levels in neutrophil bacterial killing has intriguing implications for diseases in which Cl^−^ levels in certain microenvironments may be abnormally low. For example, patients affected with cystic fibrosis are plagued with chronic pulmonary bacterial infection leading to neutrophilic inflammatory airways disease, which eventually leads to irreversible airways destruction, respiratory failure and premature death. The underlying genetic defect in cystic fibrosis is a mutation in the CFTR gene, which encodes a Cl^−^/bicarbonate ion channel located in the apical surface of airway epithelial cells. In normal airway epithelial cells, CFTR is thought to regulate airway surface liquid hydration in part by secretion of Cl^−^ into the airway surface liquid. In CF, it is thought that there is decreased Cl^−^ secretion to the airway surface liquid, and hypothetically lower Cl^−^ levels. When neutrophils are recruited to this microenvironment in the airways by chronic bacterial infection, it is possible that the abnormal ionic milieu negatively influences the ability of the neutrophil to effectively kill bacteria.

While our data clearly identifies HOCl as the key ROS in NET generation, the mechanism by which it may function in this capacity is still unknown. Previous *in* *vitro* studies have suggested that HOCl can chlorinate nuclear histone proteins [13]. Shifts in histone mobility occur upon NET stimulation [10], and citrullination (arginine deamination), another post-translational modification of histones, has been found to play a key role in the chromatin decondensation that precedes NETosis [14]. However, Papayannopoulos *et* *al*. suggest that MPO effects on nuclear decondensation may by independent of its enzymatic activity [10]. Thus our finding that MPO pharmacological inhibition blocks, while HOCl stimulates, NETosis indicates additional effects of HOCl on cell physiology relevant to the NET phenotype. For example, earlier studies have shown that HOCl can chlorinate membrane cholesterol moieties *in* *vitro* [15], [16], and that chlorination of membrane cholesterol moieties contributes to changes in membrane fluidity and integrity [17]. Thus HOCl may influence the stereotyped dissolution or disruption of multiple membrane bound compartments (cytoplasmic vesicles, nuclear envelope, and eventually cell membrane) that occurs at later stages of NET formation, an interesting area for future biophysical studies.

Finally, our comparative studies of MPO deficiency and pharmacological inhibition on NET production in humans and mice reveal at least one distinct species-specific difference in the cell physiology of this phenomenon. While the mouse has proved a valuable tool for exploring the biology of NETs in infection and inflammation models, it is important to recognize that the regulatory contributions of MPO and HOCl to the observed process appear to be markedly diminished in the murine condition.

## Materials and Methods

### Antibodies and Reagents

Cells were maintained in culture in RPMI medium (Invitrogen). Rabbit anti-human MPO antibody (Dako) was used at 1∶300 for staining human NETs. Mouse anti-histone 2A/histone 2B/DNA complex antibody (kind gift from M. Monestier, Garden State Cancer Center, NJ) was used at 1∶3000 for staining murine NETs. DAPI (Roche Diagnostics) was used at 500 ng/ml. ABAH (Fisher Scientific) was used at 100 µM. Phorbol myristate acetate (Sigma) was used at 25 nM for human peripheral neutrophils, and 100 nM for bone-marrow derived murine neutrophils. H_2_O_2_ 30% (v/v) solution (EMD) was estimated to be 8.8 M. Sodium hypochlorite 10–15% (v/v) solution (Sigma) was estimated to be 1.6 M. Neutrophil viability was assessed using Live/Dead^®^ Viability Cytotoxicity Kit for mammalian cells (Life Technologies) according to manufacturer instructions.

### Neutrophil Isolation from Peripheral Blood

Blood was obtained via venopuncture from healthy volunteers with written informed consent according to a protocol approved by the University of California San Diego Human Research Protection Program. Freshly collected heparinized blood was layered onto Polymorphprep™ (Axis-Shield), and neutrophils were isolated after centrifugation at 600×*g* for 30 min per the manufacturer’s protocol. Cells were washed in PBS, and RBC removed with hypotonic lysis in sterile water. Neutrophils were counted using a hemocytometer.

### Neutrophil Derivation from Murine Bone Marrow

WT and MPO-deficient mice in the C57BL/6 background [12] were humanely euthanized with overdose of inhaled isofluorane and cervical dislocation to ameliorate suffering, and hind legs dissected. Overlying skin and muscle were removed from femur and tibia, being careful to keep these bones intact to maintain sterility of bone marrow. Dissected legs were shipped at 4°C overnight in RPMI supplemented with 10% (v/v) fetal bovine serum, 25% (v/v) L-cell conditioned media, 2 mM L-glutamine, and penicillin/streptomycin. Using sterile tools, ends of long bones were cut to expose marrow, and the cells were flushed out with ice-cold RPMI medium. Cells were washed in PBS, and resuspended in 45% (v/v) Percoll (GE Healthcare). Neutrophils were isolated using centrifugation over a Percoll density gradient (81%/62%/55%/50%), at the interface between 81% and 62%. Cells were washed in PBS and counted on hemocytometer.

### Neutrophil Collection from Murine Skin Pouch

Wild type CD1 female mice were sedated using titrated dose of inhaled isofluorane. The dorsal fur was removed by shaving, and 5 ml of sterile filtered air was introduced under the skin of the upper back to create an air pouch, 6 d prior to ultimate neutrophil collection. The air pouch was reinflated with 3–5 ml of sterile filtered air at 3 d prior to neutrophil collection. On the day of neutrophil collection, the dorsal air pouch was injected with 600 µl of LPS (2 µg) in 2% (w/v) carboxymethycellulose (Sigma). Four h after LPS injection, mice were euthanized by overdose of inhaled isofluorane and secondary cervical dislocation. Air pouches were injected with 5 ml sterile PBS, and fluid containing recruited peripheral neutrophils was collected from the pouch.

### Microbial Killing Assay

Human peripheral blood neutrophils were washed and incubated in minimal media +2% (v/v) fetal calf serum containing Cl^−^ or no Cl^−^. Next, 2×10^5^ cells were seeded in triplicate in 96-well tissue culture plates, with wells containing media alone as controls. A liquid culture of *P. aeruginosa* (PA01) in Luria broth was grown to OD_600_ = 0.6 (∼2×10^8^ CFU/ml). Bacteria were washed ×3 in dPBS, and resuspended in minimal media containing Cl^−^or no Cl^−^, and 2×10^4^ CFU added to each well (MOI = 0.1). Plates were centrifuged at 500×*g* for 10 min to promote contact between adherent cells and bacteria, then incubated at 37°C/5% CO_2_ for 30 min. The bacterial CFU remaining at the end of the incubation period were enumerated by serial dilution and plating.

### Quantitative NET Assay

Neutrophils were stimulated as indicated in tissue culture multi-well plates. Released extracellular DNA was digested by addition of 500 mU of micrococcal nuclease (Worthington) for 10 min at 37°C with gentle agitation. The preparation was centrifuged at 200×*g* for 8 min, and the supernatant mixed 1∶1 with PICO green reagent (Quant-iT™ PICO green® dsDNA Detection Kit, Invitrogen) per the manufacturer’s protocol. Fluorescence was quantified on SpectraMax M3 plate reader, Ex 485/Em 520 using SoftMax Pro software.

### NETosis Assays Using Immunostaining and Microscopy

Neutrophils were stimulated as described in tissue-culture multi-well plates. For assays where cells were pre-treated with ABAH or vehicle control, cells were incubated in compounds at room temperature for 30 min prior to stimulation. Cells were then treated with indicated stimuli (PMA +/− bacteria) while keeping the concentration of inhibitor constant throughout experiment. During stimulation, cells were incubated at 37°C and 5% CO_2_ for indicated times. Cells were fixed using 4% (v/v) paraformaldehyde (Electron Microscopy Sciences) overnight at 4°C. Cells were washed in PBS, and incubated in blocking solution (2% (v/v) normal goat serum +2% (w/v) bovine serum albumin in PBS) for 1 h at room temperature. Cells were then incubated in primary antibody in 2% (w/v) BSA inPBS, at room temperature for 1 h (or at 4°C overnight), washed 3 times in PBS, and incubated in fluorescent-labeled secondary antibody and DAPI in 2% (w/v) BSA inPBS for 1 h at room temperature. Cells were washed ×3 in PBS prior to microscopy. Cells were visualized using a Zeiss Axiovert 40 CFL microscope, Zeiss Achroplan 10X, or Achrostigmat 32X objectives, and images collected using Zeiss Axiocam MR camera and AxioVision AC software.

### Extracellular Chlorination Assay (MPO Activity Assay)

This assay was adapted from protocol published by Kettle *et* *al* [8]. Neutrophils were incubated in RPMI medium (or specialized medium, as indicated) containing 5 mM taurine at room temperature for 15 min. Cells were stimulated as indicated, and incubated for another 30 min at room temperature. Catalase 20 ug/ml (Sigma) was then added to quench the reaction, and tubes placed on ice for 10 minutes. Cells were pelleted, and supernatant transferred to 96-well plate. Developing reagent (sodium acetate buffer pH 5.4, 2 mM TMB, 10% (v/v) DMF, 100 µM NaI) was added, and mixture incubated for 5 min. Colorimetric change measured using SpectraMax 250 spectrophotometer read at A650. Concentration of extracellular HOCl produced was determined using standard curve from known concentrations of HOCl.

### Oxidative Burst Assay

Neutrophils were incubated in HBSS (Ca^2+^ and Mg^2+^-free) with 10 µM 2,7 di-chlorofluorescein diacetate (DCF-DA, Fisher) at 37°C for 20 min with continuous rotation. Cells were then pelleted, and washed in PBS (or indicated buffers) ×3, and transferred to a 96-well plate (1×10^6^ cells/well in 100 µL volume). Cells were stimulated, and absorbance read every 15 min using SpectraMax M3 (Molecular Devices) fluorescent plate reader at excitation 485 nm, emission 530 nm.

### Statistical Analysis

Statistical analyses were done using GraphPad Prism software. Standard error bars are shown where quantitative data collected for experimental conditions were done in triplicate. All experiments were done least 3 independent times to ensure repeatability of results (unless otherwise stated), and representative data are shown. Statistical significance was performed using 2-way ANOVA analysis of experimental groups if more than two groups were analysed, and student t-test (unpaired) if one group is compared to a control group.

### Ethics Statement

Blood was obtained via venopuncture from healthy volunteers under written informed consent in protocol #080451 approved the University of California San Diego (UCSD) Human Research Protection Program. Isolation of neutrophils from murine bone marrow and skin pouch was performed according to the approved UCSD Animal Care and Use Committee protocol S00227M.
